# An Effective Way for Simulating Oceanic Turbulence Channel on the Beam Carrying Orbital Angular Momentum

**DOI:** 10.1038/s41598-019-50465-w

**Published:** 2019-09-30

**Authors:** Sunxiang Pan, Le Wang, Wennai Wang, Shengmei Zhao

**Affiliations:** 1 Institute of Signal Processing and Transmission, Nanjing University of Posts and Telecommunications(NUPT), Nanjing, 210003 China; 2 Key Lab of Broadband Wireless Communication and Sensor Network Technology, Ministry of Education, Nanjing, 210003 China

**Keywords:** Quantum optics, Quantum optics

## Abstract

In this paper, we present an effective way for simulating oceanic turbulence channel on the beam carrying orbital angular momentum (OAM). The influence caused by oceanic turbulence channel on the phase and intensity of the propagation beam is equivalent to that the beam passing through several individual phase screens generated by power spectrum inversion method at regular intervals. A modified subharmonic compensation method is then further balance the phase screen for the losses of lower frequency components in the power spectrum inversion method. The feasibility is verified by the theoretical phase structure function and the propagation characteristics of an OAM beam in underwater environment. The results show that the phase structure function and the propagation characteristics of the OAM beam evaluated by the phase screen model all coincide with those theoretical results at high spatial frequency. Simultaneously, the low frequency components could be effectively compensated by the modified subharmonic method. With the increase of the subharmonic order and sample level, the performance evaluated by the phase screen model are closer to the theoretical ones. It has provided an effective way for simulating oceanic turbulence channel for the underwater optical communications.

## Introduction

Since a relatively low attenuation in blue-green range (450–550 nm) was found for optical communications in an underwater environment^1^, underwater wireless optical communication (UWOC) has attracted much attentions^2^. UWOC lends an advantage of broader bandwidths and a potential for higher levels of security compared to its acoustic counterpart^3^. Besides, UWOC can include multiplexing schemes that further increase the capacity, where the known multiplexing scheme is space division multiplexing (SDM), involving spatially modifying different data-encoded light sources for co-aligned propagation and subsequent receiver discrimination and retrieval.

One form of SDM in free space optical communications that has recently received much attention is orbital angular momentum (OAM)^4–8^. A light beam with a helical wavefront carries an OAM value corresponding to $\ell \hslash$ per photon, where $\hslash$ is the reduced Planck’s constant and $\ell$ is an unbounded integer that represents the number of 2*π* phase changes in the azimuthal direction. Due to different OAM modes are inherently orthogonal^9^, the OAM-multiplexed optical communications can handle a huge amount of information and tremendously increase the capacity of communication link^4–8^.

Very recently, OAM multiplexing technique has been introduced to UWOC^10–14^. For example, J. Baghdady *et al*. reported 3-Gbit/s UWOC employing 2 OAM modes multiplexing^10^. Y. Ren *et al*. further increased the UWOC transmission capacity to 4 Gbit/s (directly modulated LD) and 40 Gbit/s (external modulation & frequency doubling) by multiplexing 4 green OAM modes^11^. Also 2-meter underwater transmission of 4-fold green light (520 nm) OAM modes was demonstrated for a UWOC multicasting link in^12^.

At the same time, many theoretic research works on the evolution of optical beam carrying OAM in the underwater environment have been reported^15–19^. For instance, Xu *et al*.^15^ deduced the expression of the cross-spectral density matrix for the propagation of random electromagnetic vortex beams in oceanic turbulence, while Cheng *et al*.^16^ used Rytov approximation theory to calculate the influence of ocean turbulence on the transmission of Laguerre-Gaussian (LG) beam.

For all the experimental results^10–14^, the underwater conditions are emulated by using a fixed length tank filled with tap water, and only limited underwater conditions, such as, scattering/turbidity, current, and thermal gradients could be setup; For the theoretical results^15–19^, the analytical solutions are always derived by the complex mathematical calculation on beam expression with Rytov approximation method. The derivations are generally complicated and sometimes there are no analytical solutions. It is desirable to have an easy way to simulate the optical propagating in underwater channel for exploring the performance of a UWOC system or a UWOC system carrying OAM.

In the paper, we present an effective way for simulating oceanic turbulence channel on the propagation beam carrying orbital angular momentum (OAM) using power spectrum inversion method. The influence caused by underwater channel on the intensity and phase of the propagation beam is equivalent to the propagation results when the beam passes through several individual phase screens at regular intervals. At the same time, a modified subharmonic compensation method is used to solve the low frequency loss problem in a large-scale phase fluctuations. The oceanic fluctuation spectrum in the index of refraction proposed by Nikishov *et al*.^20^ is adopted in the model. The theoretic phase structure function and the theoretic propagation characteristics of an OAM beam in oceanic turbulence are utilized to verify the proposed method.

## Random Phase Screens Model for Simulating Oceanic Turbulence Channel

In the section, we present the random phase screens model to simulate the beam propagating through the underwater environment. Several individual phase screens generated by power spectrum inversion method at regular intervals is utilized to simulate the influence of ocean turbulence on the propagation beam.

### The random phase screens model

Oceanic turbulence environment is a complex system of physical, chemical and biological combinations. The effect of these factors on beam propagation can ultimately be attributed to kinetic energy and temperature gradients, which will caused different refraction index in different regions. Due to the fluctuations in density, salinity and temperature of the oceanic turbulence environment, the variation of the refraction index along the propagation path would lead to the beam intensity fluctuations in a random manner. These intensity fluctuations would then severely influenced beam propagation properties, like intensity distribution, beam wander and scintillation index, and finally degrade the performance of an UWOC system. Hence, an useful optical beam propagation model for oceanic turbulence is necessary for predicting the performance of the UWOC system.

**Figure 1:**
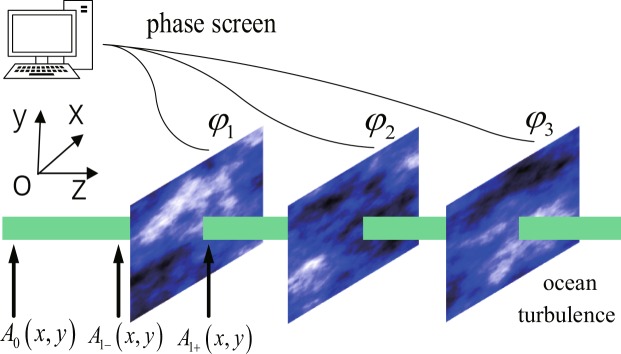
The schematic diagram of random phase screens model for simulating oceanic turbulence. The phase screen is in the *x* − *y* plane, and the direction of beam propagation is in *z*-axis.

Here, we use successive phase screens at regular intervals to represent the oceanic turbulence, named random phase screens model^21–23^, which is numerical simulation of the light field where the continuous random media is represented as a series of random phase screens transverse to the propagation direction. In the model, multiple individual phase screens are introduced to the beam with a regular interval. Each phase screen is a thin sheet that adjusted the phase of the beam. The perturbation effects, such as those induced by oceanic turbulence, could be incorporated by a split-step method which treating propagation and phase perturbations separately in discrete steps along the propagation axis. In each step, the phase perturbations caused by the refractive index fluctuation arising from turbulence are implemented by multiplying the input field with a phase exponential function (a phase screen), and then the beam undergoes a free-space propagation between the adjacent two phase screens. Additionally, the phase screens are statistically independent of the spatial statistics that matched the index of refraction fluctuations. Figure 1 is the schematic diagram of the oceanic turbulence phase screens model, the phase screen is in the *x* − *y* plane, and the direction of beam propagation is in *z*-axis.

Consider an OAM beam in the plane transmitter (*z* = 0), the initial beam in the spatial domain (the beam field) is *A*_0_(*x*, *y*, *z* = 0), where *x*, *y* are spatial domain coordinates at *z* = 0. Before the first phase screen, the beam field should be ${A}_{{1}_{-}}(x\text{'},y\text{'},z)$ according to the Fresnel diffraction formula.

1 $${A}_{{1}_{-}}(x\text{'},y\text{'},z)=\frac{i\,\exp \,(\,-\,ikz)}{\lambda z}\int {\int }_{-\infty }^{\infty }\,{A}_{0}(x,y,z=0)\,\exp \,\{-i\frac{k}{2z}[{(x\text{'}-x)}^{2}+{(y\text{'}-y)}^{2}]\}dxdy,$$

where $i=\sqrt{-1}$, is the imaginary unit, *x*′, *y*′ are the spatial domain coordinates at *z* = Δ*z* (Δ*z* is the transmission distance between the transmitter to the first phase screen), *λ* is the wavelength, *k* = 2*π/λ* is the wave number (wave vector). After the beam passes through the first phase screen *φ*_1_(*x*, *y*), the random phase would impress on the beam’s field, and the beam field turns to

2 $${A}_{{1}_{+}}(x,y,z{|}_{z=\Delta z})={A}_{{1}_{-}}(x,y,z{|}_{\Delta z})\,\exp \,[i{\phi }_{1}(x,y)],$$

The phase screen *φ*_1_(*x*, *y*) in the simulation may be expressed as a *N*_*x*_ × *N*_*y*_ array of random complex numbers, whose statistics match those fluctuations of oceanic turbulence.

This procedure is then repeated for the second phase screen by replacing *A*_0_(*x*, *y*, *z*) with ${A}_{{1}_{+}}(x,y,z)$, replacing ${A}_{{1}_{+}}(x,y,z)$ with ${A}_{{2}_{-}}(x,y,z)$ in Eq. (1), and replacing ${A}_{{1}_{+}}(x,y,z)$ with ${A}_{{2}_{+}}(x,y,z)$, replacing ${A}_{{1}_{+}}(x,y,z)$ with ${A}_{{2}_{-}}(x,y,z)$, *φ*_1_(*x*, *y*) with *φ*_2_(*x*, *y*) in Eq. (2). The process should be repeated until the last phase screen is reached.

The above phase screens *φ*_1_(*x*, *y*), *φ*_2_(*x*, *y*), …, *φ*_M_(*x*, *y*) can be produced by filtering a white Gaussian noise with a phase screen spectrum, followed by Fourier transform. Generally, the relation between the phase screen spectrum *F*_*φ*_(*k*_*x*_, *k*_*y*_) and the spectrum of refractive-index fluctuations spectrum of the refractive index variations Φ(*k*_*x*_, *k*_*y*_) can be described as

3 $${F}_{\phi }({k}_{x},{k}_{y})=2\pi {k}^{2}\Delta z\Phi ({k}_{x},{k}_{y}),$$

where *k* = 2*π*/*λ* is the wave vector, Δ*z* is the spacing distance between the subsequent phase screens.

Therefore, a phase screen in simulation $\phi (j\Delta x,l\Delta y),\,(0\le j\le {N}_{x},\,0\le l\le {N}_{y})$ with size *L*_*x*_ = *N*_*x*_ × Δ*x*, *L*_*y*_ = *N*_*y*_ × Δ*y* (*N*_*x*_ and *N*_*y*_ are the array sizes of phase screen, Δ*x* and Δ*y* are the spatial-sampling intervals at *x*, *y* directions), is then described as

4 $$\begin{array}{rcl}\phi (j\Delta x,l\Delta y) & = & {\rm{FFT}}\,[a(m,n)+ib(m,n)]\\  & = & \mathop{\sum }\limits_{m=0}^{{N}_{x}}\,\mathop{\sum }\limits_{n=0}^{{N}_{y}}\,[a(m,n)+ib(m,n)]\,\exp \,[2\pi i\,(jn/{N}_{x}+lm/{N}_{y})].\end{array}$$

where *i* is also the imaginary unit, *a*(*m*, *n*), *b*(*m*, *n*) are zero mean Gaussian uncorrelated random numbers, which are described as

5 $$\langle {a}^{2}(m,n)\rangle =\langle {b}^{2}(m,n)\rangle =\Delta {k}_{x}\Delta {k}_{y}{F}_{\phi }(m\Delta {k}_{x},n\Delta {k}_{y}).$$

Here, 〈·〉 denotes ensemble average, Δ*k*_*x*_, Δ*k*_*y*_ are the spectrum-sampling intervals, Δ*k*_*x*_ = 2*π*/*L*_*x*_, Δ*k*_*y*_ = 2*π*/*L*_*y*_, respectively. *a*(0, 0) = *b*(0, 0) = 0, since the zero frequency component does not change the spatial statistics of the fields.

The refractive index spectrum Φ(*k*) for homogeneous and isotropic oceanic turbulence could be expressed as^20^,

6 $$\Phi (k)=0.388\,{C}_{n}^{2}{k}^{-11/3}[1+2.35{(\eta k)}^{2/3}]({\omega }^{2}{e}^{-{A}_{T}\delta }+{\omega }^{-2}{e}^{-{A}_{S}\delta }-2{\omega }^{-1}{e}^{-{A}_{TS}\delta })$$

where *k* is the wave vector, ${C}_{n}^{2}={10}^{-8}{\varepsilon }^{-1/3}{\chi }_{T}$ is the refractive-index structure constant, *ε* is the rate of dissipation of kinetic energy per unit mass of fluid (ranging from 10^−1^ *m*^2^/*s*^3^ to 10^−10^ *m*^2^/*s*^3^), *η* is the Kolmogorov micro scale (inner scale), *χ*_*T*_ is the rate of dissipation of mean-squared temperature (ranging from 10^−4^ *K*^2^/*s* to 10^−10^ *K*^2^/*s*), *ω* is the ratio of temperature and salinity contributions to the refractive index spectrum (varying from −5 to 0 for oceanic turbulence, where −5 represents the dominating temperature-induced, 0 denotes the salinity-induced optical turbulence), *A*_*T*_ = 1.863 × 10^−2^, *A*_*S*_ = 1.9 × 10^−4^, *A*_*TS*_ = 9.41 × 10^−3^, and *δ* = 8.284(*kη*)^3/4^ + 12.978(*kη*)^2^.

### The modified subharmonic compensation method

The split-step method has the advantages of much faster speed and smaller memory requirements, and is suitable for generating large-scale phase screen. However, it suffers from a drawback of the under-sampling for the low spatial frequency components. Near the origin in Fourier space, the spectrum is changing so rapidly that the sampling near the origin is not enough to simulate all low frequency components, especially those frequencies with periods greater than the size of phase screen, such as, the spectral region (−Δ*k*_*x*_/2, Δ*k*_*x*_/2) and (−Δ*k*_*y*_/2, Δ*k*_*y*_/2), which would result in an inadequate simulation of the ocean turbulence random phase screen in the large-scale phase fluctuations.

**Figure 2:**
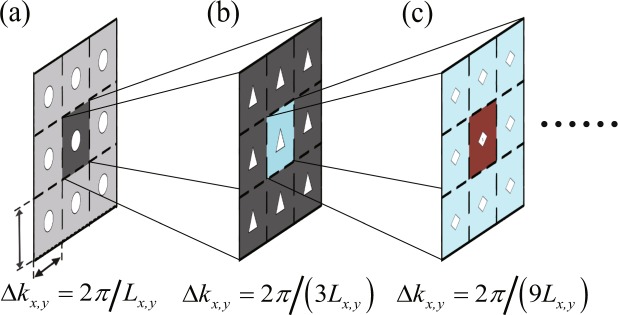
The diagram for the modified subharmonic compensation method. Here, the sample level is 3 × 3. (**a**) The sampled value, (**b**) the first subharmonic order, (**c**) the second subharmonic order. By re-sampling the origin of the power spectrum of the phase screen, the mid area is then subdivided into smaller and smaller subharmonic samples.

By re-sampling the spectrum near the origin (the central point of phase screen), the subharmonic compensation method can balance the loss of low frequency components inherent in power spectrum inversion method. Here, a modified subharmonic compensation method is presented. Figure 2 shows the modified subharmonic compensation method, denoted by the subharmonic order *p* and the sample level *q*, and the spectral-sampling interval would now turn to Δ*k*_*x*(*y*)_ = 2*π*/(*q*^*p*^*L*_*x*(*y*)_). Figure 2(a) denotes the subharmonic compensation method for 3 × 3 sample level. Figure 2(b) shows the first order subharmonic compensation in this sample level. The mid area in Fig. 2(a) has now been divided into nine sub areas, and there is 8 new interpolated spectral samples there. Figure 2(c) shows the second order subharmonic compensation in this sample level, the mid area in Fig. 2(b) has further been divided into nine smaller areas.

For the 3 × 3 sample level, there are 8 new interpolated values for the first subharmonic order compensation, and the second subharmonic order compensation is operated on the first compensation result, therefore, there exists 16 new interpolated low-frequency samples in the second subharmonic order compensation. The interpolated low-frequency samples are increased when the subharmonic order increase. The more the compensation order is, the more samples at the central point of the power spectrum has, and the more fruitful the low frequency components the phase screen has.

On the other hand, the mid area is divided into 25 smaller areas for the first subharmonic order compensation at 5 × 5 sample level, and there exists 24 new interpolated samples for the first subharmonic order compensation. Continually, the mid area is then divided into 49 smaller areas for the first subharmonic order at 7 × 7 sample level, and there exists 48 new interpolated samples for the first subharmonic order. When the sample level increase, the new interpolated subharmonic samples values grow up quickly.

With the modified subharmonic compensation, the phase screen with low frequency components can be subsequently expressed as^24^,

7 $$\begin{array}{rcl}{\theta }_{sh}(j\Delta x,l\Delta y) & = & \mathop{\sum }\limits_{p=1}^{{N}_{p}}\,\mathop{\sum }\limits_{s=-\,1}^{1}\,\mathop{\sum }\limits_{t=-\,1}^{1}\,[a(s,t,p)+ib(s,t,p)]\\  &  & \times \exp \,[2\pi i\,(js/({q}^{p}{N}_{x})+lt/({q}^{p}{N}_{y}))]\\  & = & {\rm{FFT}}\,[a(s,t,p)+ib(s,t,p)]\end{array}$$

where *N*_*p*_ is the given subharmonic order, *p* is a subharmonic order variant (from 1 to *N*_*p*_), *s*, *t* are variants *s*, *t* ∈ [−1, 0, 1]. The spectral grid sizes for the subharmonic order *p* turn to Δ*k*_*xp*_ = 2*π*/(*q*^*p*^*L*_*x*_), Δ*k*_*yp*_ = 2*π*/(*q*^*p*^*L*_*y*_). Here *q* × *q* is the sample level of the modified subharmonic compensation. When *q* = 3 the Eq. 7 falls back to the techniques proposed by Lane *et al*.^25^ based on the addition of subharmonics with random complex amplitudes to improve the large-scale statistics. *a*(*s*, *t*, *p*), *b*(*s*, *t*, *p*) are also zero mean Gaussian uncorrelated random numbers, they are described as

8 $$\langle {a}^{2}(s,t,p)\rangle =\langle {b}^{2}(s,t,p)\rangle =\Delta {k}_{xp}\Delta {k}_{yp}{F}_{\Phi }(s\Delta {k}_{xp},t\Delta {k}_{yp})$$

Here, the spectrum of the phase screen is

9 $${F}_{\Phi }(s\Delta {k}_{xp},t\Delta {k}_{yp})=2\pi {k}^{2}\Delta z\Phi \,(s\Delta {k}_{xp},t\Delta {k}_{yp},{k}_{z}=0).$$

In the simulation, the compensated sample values are first subjected to discrete Fourier transform to obtain the added phase distribution generated by the modified subharmonic compensation, and then they are added to the random phase screen created by using power spectrum inversion method. The final phase screen can be represented as the superposition of the initial phase screen and the compensated phase screen, *θ* = *φ* + *θ*_*sh*_.

## Model Verification and Validation

In this section, we verify the validity of the model from two aspects. One is the phase structure function, the other is the propagation property of beam carrying OAM modes through the underwater environment.

### Phase structure function

It is shown the mean squared difference for any two points in the index of refraction only depends on the distance between the two points, with no relation to the direction in Kolmogorov model^26^. Hence, the statistics characteristics of the underwater turbulence can be described by a structure function.

According to the results in^27^, the theoretical phase structure function of a plane wave was described as

10 $$D(r)=\{\begin{array}{ll}2{(r/{r}_{0})}^{2}, & r\ll \eta \\ 2{(r/{r}_{0})}^{5/3}, & r\gg \eta \end{array},$$

where *r*_0_ was a plane-wave spatial coherence radius, defined as the separation distance at which the modulus of the complex degree of coherence fell to 1/*e*. Here, the plane-wave spatial coherence radius is

11 $${r}_{0}=\{\begin{array}{l}{[3.603\times {10}^{-7}{k}^{2}{\varepsilon }^{-1/3}\frac{{\chi }_{T}}{2{\omega }^{2}}\times (16.958{\omega }^{2}-44.175\omega +118.923)]}^{-1/2},\,r\ll \eta \\ {[3.603\times {10}^{-7}{k}^{2}{\varepsilon }^{-1/3}\frac{{\chi }_{T}}{2{\omega }^{2}}\times (1.116{\omega }^{2}-2.235\omega +1.119)]}^{-3/5},\,r\gg \eta \end{array},$$

where *ε*, *χ*_*T*_, *ω* are defined as Eq. 6. Equation 10 indicates that under Rytov approximation, the Kolmogorov five-thirds power law of wave structure function is also valid for oceanic turbulence in the inertial range if the power spectrum of oceanic turbulence proposed by Nikishov is adopted.

On the other hand, we also can evaluate the phase structure function with the beam field simulated under the random phase screens model. Therefore, the phase structure function can be evaluated as

12 $$D(r)=\langle {[\phi (r)-\phi (r\text{'})]}^{2}\rangle ,$$

where 〈·〉 is an ensemble average, *φ*(*r*) is the field amplitude at *r* point, *r*, *r*′ are the two points in the phase screen.

To compare the evaluated phase structure function with the theoretical one, we used 1000 random phase screens to calculate the assemble average in the numerical simulations. Simultaneously, the oceanic turbulence simulation parameters were set as following. *ε* = 0.01 *m*^2^/*s*^3^, *χ*_*T*_ = 10^−10^ *K*^2^/*s*, *ω* = −4, *η* = 0.0001, *z* = 50 m. The wavelength of the beam was 488 *nm*. The size of the phase screen was 0.8 *m* × 0.8 *m* and the sampling points were 81 × 81.

**Figure 3:**
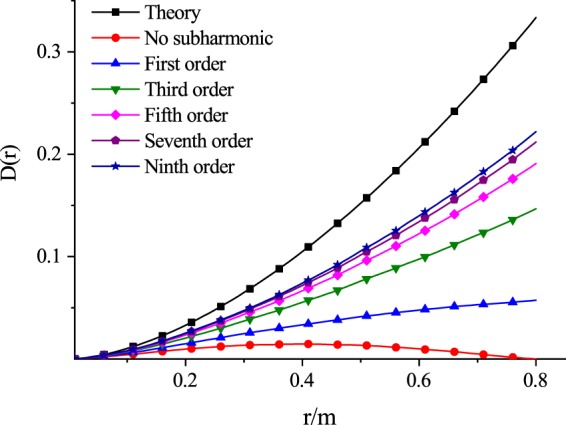
The phase structure function calculated under the random phase screens model, the theoretical phase structure function with Eq. 10, and those results with the modified subharmonic compensation method.

Figure 3 shows the phase structure function evaluated under the random phase screens model, the theoretical phase structure function with Eq. 10, and those results with the modified subharmonic compensation method. The oceanic turbulence simulation parameters were set as *ε* = 0.01 *m*^2^/*s*^3^, *χ*_*T*_ = 10^−10^ *K*^2^/*s*, *ω* = −4, *η* = 0.0001, *z* = 50 m. The modified subharmonic compensation method had 3 × 3 sample level, and the subharmonic order was the first order, the second order and the third order. The results showed that the phase structure function of phase screens was very close to the theoretical one (*r* ≫ *η*), when the distance between two points was smaller, say, less than 10% of the size of phase screen; With the distance increases, the difference between the theory one and the evaluated one increased. When the distance between two points on the phase screen was close to the size of the phase screen, the evaluated structure function (red curve) dropped very quickly, was far from the theoretical value (black curve).

On the other hand, the evaluated phase structure function from the improved phase screens model had better performance than that one without compensation. As the more and more subharmonic components were added to phase screens model, the evaluated phase structure function approached the theoretical one, which indicated that the subharmonic compensation method could effectively overcome the lack of low frequency components problem in power spectrum inversion method.

We further discuss the effect of the sample level on the compensation. In order to analyze the compensation effect, we define a parameter *CE* to qualify the proximity between two curves.

13 $$CE=\frac{{\sum }_{i}\,|f(i)-{f}_{nosub}(i)|}{{\sum }_{i}\,|{f}_{theory}(i)-{f}_{nosub}(i)|}$$

where *f*(*i*) denotes the evaluated phase structure function value when *r* = *i*, *f*_*nosub*_(*i*) denotes the evaluated phase structure function value without compensation, *f*_*theory*_(*i*) means the theory phase structure function value. *i* ∈ [0, 2] are some discrete sample values of *r*. *CE* ∈ [0, 1]. The higher *CE*, the closer the evaluated phase structure function values to the theoretical one, indicating the better compensation effect.

**Figure 4:**
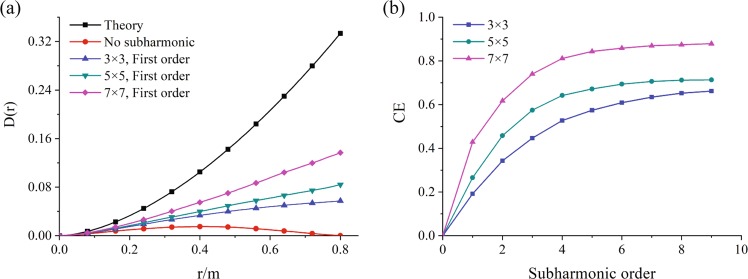
The compensation effect with different subharmonic order (*p*) and sample level (*q*).

Figure 4 shows the compensation effect with different subharmonic order (*p*) and sample level (*q*), where Fig. 4(a) illustrates the phase structure function with different sample level, Fig. 4(b) shows the compensation effect against the sample level. Simultaneously, the oceanic turbulence simulation parameters were set as same as that in Fig. 3, *ε* = 0.01 *m*^2^/*s*^3^, *χ*_*T*_ = 10^−10^ *K*^2^/*s*, *ω* = −4, *η* = 0.0001, *z* = 50 m. The results showed that the evaluated phase structure function was approaching to the theoretical one when the sample level (*q*) was increased. For the lower subharmonic order, the compensation effect *CE* increased quickly with the sample level. However, the compensation effect became constants when the subharmonic order was greater than 5. The higher sample level was, the better the compensation effect was.

**Figure 5:**
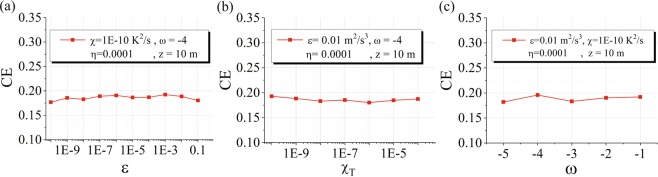
The influence of turbulence parameters on the compensation effect.

Figure 5 further discusses the influence of turbulence parameters in phase screens model on the compensation effect. Figure 5(a–c) describe the compensation effect against the rate of dissipation of kinetic energy *ε*, the rate of dissipation of mean-squared temperature *χ*_*T*_, and the ratio of temperature and salinity contributions to the refractive index spectrum *ω*, respectively. The results showed that the compensation effect varied little with these parameters (*ε*, *χ*_*T*_ and *ω*), which indicated that the random phase screens model had a good stability and was available for simulating the oceanic turbulence.

### Propagation characteristics of an OAM beam

In this subsection, we further verify the proposed random phase screens model by the propagation characteristics of an OAM beam in oceanic turbulent environment. Here, the Laguerre-Gauss(LG) mode was selected as the OAM beam candidate. The electric field of LG beam in *z*-axis direction in the cylindrical polar coordinate *U*(*r*,*φ*, z) is expressed as,

14 $$U(r,\phi ,z)=\sqrt{\frac{2m!}{\pi (m+|\ell |)!}}\frac{1}{\omega (z)}{[\frac{r\sqrt{2}}{\omega (z)}]}^{|\ell |}{{\rm{L}}}_{m}^{\ell }[\frac{2{r}^{2}}{{\omega }^{2}(z)}]\,\exp \,[\frac{-ik{r}^{2}z}{2({z}_{R}^{2})}]\,\exp \,[i(2m+|\ell |+1){\tan }^{-1}\frac{z}{{z}_{R}}]\,\exp \,(i\ell \phi )],$$

where *m* is the number of radial nodes in the intensity distribution, *l* is topological charge, giving an OAM of $\ell \hslash$ per photon, ${z}_{R}=\pi {\omega }_{0}^{2}/\lambda$ is Rayleigh distance, $\omega (z)={\omega }_{0}\sqrt{1+{z}^{2}/{z}_{R}^{2}}$ is the beam width*, ω*_0_ is beam waist, ${{\rm{L}}}_{m}^{\ell }[\cdot ]$ is an associated Laguerre polynomial.

When a LG beam carrying topological charge ${\ell }_{0}$ passes through the oceanic turbulence, not only the intensity of beam, but also its phase distribution will change. OAM is no longer pure and its energy will spread to the adjacent OAM states. The crosstalk would occur between different OAM modes, and the resultant beam can be considered as a superposition of all OAM modes. Since OAM modes with different topological charges are mutually orthogonal, at the receiver, the intensity of $({\ell }_{0})$ OAM state can be obtained by using an inverse spiral phase mask $\exp (\,-\,i{\ell }_{0}\phi )$ since the resultant beam has turned back to Gaussian mode. Moreover, the intensity for the detection probability of each OAM state should be normalized. Hence, the probability of mode $({\ell }_{0})$ in the resultant beam in theory can be detected as

15 $${p}_{{\ell }_{0}}(z)=\frac{{\int }_{0}^{+\infty }\,\langle |{U}_{{\ell }_{0}}(r,\phi ,z){|}^{2}\rangle \,rdrd\phi }{{\sum }_{\ell }\,{\int }_{0}^{+\infty }\langle |{U}_{\ell }(r,\phi ,z){|}^{2}\rangle \,rdrd\phi },s.t.\,\sum _{\ell }\,{p}_{\ell }(z)=1$$

where $\langle |{U}_{{\ell }_{0}}(r,\phi ,z){|}^{2}\rangle$ is the assemble average of intensity for $\ell ={\ell }_{0}$ mode at the detection, which in^28^ was deduced as

16 $$\langle |{U}_{{\ell }_{0}}(r,\phi ,z){|}^{2}\rangle =\frac{4{m}_{0}!}{{\omega }^{2}(z)({m}_{0}+|{\ell }_{0}|)!}{({{\rm{L}}}_{{m}_{0}}^{{\ell }_{0}}[\frac{2{r}^{2}}{{\omega }^{2}(z)}])}^{2}{(\frac{2{r}^{2}}{{\omega }^{2}(z)})}^{{\ell }_{0}}\times \exp \,(-\frac{2{r}^{2}}{{\omega }^{2}(z)}-\frac{2{r}^{2}}{{\tilde{r}}_{0}^{2}}){{\rm{I}}}_{\ell -{\ell }_{0}}(\frac{2{r}^{2}}{{\tilde{r}}_{0}^{2}})$$

where I_*n*_(⋅) was *n* order Bessel function of the first kind, ${\tilde{r}}_{0}$ was the effective spatial coherence length^16^, which can be expressed as ${\tilde{r}}_{0}={a}_{s}^{0}{\rho }_{0}$ with ${a}_{s}^{0}={(1+{\rho }_{0}^{2}/{\rho }_{s}^{2})}^{-1/2}$. ${\rho }_{s}^{2}$ is the effects of spatial coherence of the source and ${\rho }_{0}^{2}$ is the coherence length of phase fluctuations, where ${\rho }_{0}={[{\pi }^{2}{k}^{2}z/3{\int }_{0}^{\infty }{k}^{3}\Phi (k)]}^{-1/2}$. Φ(*k*) is oceanic refractive index spectrum, which is already expressed in Eq. 6. After obtaining the light field of LG beam, it is convenient to archive some beam propagation properties, such as beam wander, scintillation index, etc.

**Figure 6:**
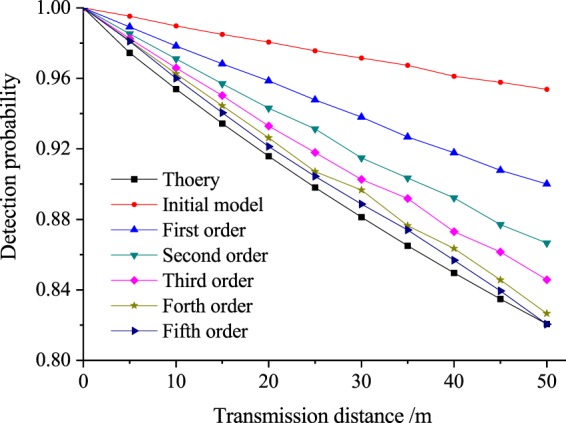
The detection probability performance of an OAM beam propagating through the oceanic turbulence verses propagation distance. The subharmonic compensation method (first order to fifth order) was used in random phase screen model. The bottom black curve was the theoretical one.

Of course, we can obtain the probability distribution of the OAM beam on ${\ell }_{0}$ mode in oceanic turbulence by the calculated electric field of LG beam after the proposed random phase screens. Figure 6 shows the detection probability of LG beam with ${\ell }_{0}$ mode versus the beam propagation distance with the calculated LG electric field, where the propagation distance varied from 1 ~ 50 m, ${\ell }_{0}=1$, *p*_0_ = 0, the waist of LG beam was 0.01 m, and the beam wavelength was 488 nm, the size of the phase screen was 0.8 m × 0.8 m with 81 × 81 points, the number of the phase screens was 5. The parameters for the proposed random phase screen simulating oceanic turbulence were the following, *ε* = 0.001 m^2^/s^3^, *χ*_*T*_ = 10^−8^ K^2^/s, *ω* = −4, *η* = 0.001. The detection probability was an average over 2000 times. The results showed that the detection probability calculated using the proposed random phase screens model without compensation (red curve) was a little far from the theoretical result in Eq. 16, while the detection probability was close to the theory result when the improved random phase screens model with higher subharmonic order was used. The higher the subharmonic order we used, the closer to the theory result the detection probability had. Here, the sample level was 3 × 3.

## Discussion

A random phase screens model for simulating underwater oceanic turbulence channel has been proposed in the paper based on the oceanic turbulent refractive index power spectrum. The influence caused by oceanic turbulence on the intensity and phase of the propagation beam has been equivalent to the propagation results when the beam passes through several individual random phase screens at regular intervals. In order to overcome the shortage of lower frequency components in power spectrum inversion method, a modified subharmonic compensation method has been presented to balance the lower spatial frequency loss. The proposed random phase screens model has been verified and validated by theoretical phase structure function and theoretical propagation performance of OAM beam in underwater environment. The availability can also be demonstrated by some beam propagation properties, like beam wander, scintillation index, after obtaining the light field distribution. It has been shown that all the evaluated results with the proposed phase screens model have satifiied with those theoretical ones when the random phase screen model has been improved by a higher subharmonic order and a higher sample level. The proposed random phase screens model would provide a promising way to simulate oceanic turbulence for studying UWOC system.
