# Author Correction: An Effective Way for Simulating Oceanic Turbulence Channel on the Beam Carrying Orbital Angular Momentum

**DOI:** 10.1038/s41598-020-58156-7

**Published:** 2020-01-22

**Authors:** Sunxiang Pan, Le Wang, Wennai Wang, Shengmei Zhao

**Affiliations:** 1 Institute of Signal Processing and Transmission, Nanjing University of Posts and Telecommunications(NUPT), Nanjing, 210003 China; 2 Key Lab of Broadband Wireless Communication and Sensor Network Technology, Ministry of Education, Nanjing, 210003 China

Correction to: *Scientific Reports* 10.1038/s41598-019-50465-w, published online 30 September 2019

This Article contains multiple errors in the Reference list.

Reference 8 is incorrectly listed as “Zou, L., Wang, L., Zhao, S. & Chen, H. Turbulence mitigation scheme based on multiple-user detection in an orbitalangular-momentum multiplexed system. *Chin. Phys. B*. **25**, 316–323 (2016)”. The correct reference 8 should read:

“Zou, L., Wang, L., Zhao, S. & Chen, H. Turbulence mitigation scheme based on multiple-user detection in an orbital angular-momentum multiplexed system. *Chin. Phys. B*. **25**, 114215 (2016)”.

Reference 10 is incorrectly cited as “Cochenour, B. M. *et al*. Multi-gigabit/s underwater optical communication link using orbital angular momentum multiplexing. *Opt. Express* **24**, 9794–9805 (2016)”. The correct reference 10 should read:

“Baghdady, J. *et al*. Multi-gigabit/s underwater optical communication link using orbital angular momentum multiplexing. *Opt. Express* **24**, 9794–9805 (2016)”.

Reference 12 is incorrectly cited as “Wang, A. *et al*. Demonstration of data-carrying orbital angular momentum-based underwater wireless optical multicasting link. *Opt. Express* **25**, 28743–28751 (2017)”. The correct reference 12 should read:

“Zhao, Y. *et al*. Demonstration of data-carrying orbital angular momentum-based underwater wireless optical multicasting link. *Opt. Express* **25**, 28743–28751 (2017)”.

Reference 14 is incorrectly listed as “Karahroudi, M. K. *et al*. Performance evaluation of perfect optical vortices transmission in an underwater optical communication system. *Appl. Opt*. **57**, 9797 (2018)”. The correct reference 14 should read:

“Karahroudi, M. K. *et al*. Performance evaluation of perfect optical vortices transmission in an underwater optical communication system. *Appl. Opt*. **57**, 9148–9154 (2018)”.

Additionally, reference 28 is incorrectly listed as “Hu, T., Pan, S., Wang, L. & Zhao, S. Influence of underwater turbulence on channel capacity of orbital angular momentum communication system. *Chin. J. Quant. Elect*. **35**, 499–506 (2018)”. The correct reference 28 should read:

“Hu, T., Pan, S., Wang, L. & Zhao, S. Influence of underwater turbulence on channel capacity of orbital angular momentum communication system (in Chinese). *Chin. J. Quant. Elect*. **35**, 499–506 (2018)”.

